# Rush Medical College

**Published:** 1852-11

**Authors:** 


*Rush Medical College.*

The introductory lecture in this institution was given by Dr. Brainard, Professor of Surgery, and was listened to by a large and intelligent class of students. The prospects of the college are flattering, and indicate that the efforts of the faculty to meet the wants of the profession, are appreciated. Clinical lectures are delivered in the two hospitals, by Professors Brainard, Herrick, and Davis, thus affording an opportunity for the study of practical medicine and surgery, unsurpassed by any institution in the West.

